# Efficacy Evaluation of Subtotal and Total Gastrectomies in Robotic Surgery for Gastric Cancer Compared with that in Open and Laparoscopic Resections: A Meta-Analysis

**DOI:** 10.1371/journal.pone.0103312

**Published:** 2014-07-28

**Authors:** Liang Zong, Yasuyuki Seto, Susumu Aikou, Takamasa Takahashi

**Affiliations:** 1 Department of Gastrointestinal Surgery, Graduate School of Medicine, University of Tokyo, Tokyo, Japan; 2 Division of Surgical Oncology, Department of Surgery, Nagoya University Graduate School of Medicine, Nagoya, Japan; Clínica Universidad de Navarra, Spain

## Abstract

**Purposes:**

Robotic gastrectomy (RG), as an innovation of minimally invasive surgical method, is developing rapidly for gastric cancer. But there is still no consensus on its comparative merit in either subtotal or total gastrectomy compared with laparoscopic and open resections.

**Methods:**

Literature searches of PubMed, Embase and Cochrane Library were performed. We combined the data of four studies for RG *versus* open gastrectomy (OG), and 11 studies for robotic RG *versus* laparoscopic gastrectomy (LG). Moreover, subgroup analyses of subtotal and total gastrectomies were performed in both RG *vs.* OG and RG *vs.* LG.

**Results:**

Totally 12 studies involving 8493 patients met the criteria. RG, similar with LG, significantly reduced the intraoperative blood loss than OG. But the duration of surgery is longer in RG than in both OG and LG. The number of lymph nodes retrieved in RG was close to that in OG and LG (WMD = −0.78 and 95% CI, −2.15−0.59; WMD = 0.63 and 95% CI, −2.24−3.51). And RG did not increase morbidity and mortality in comparison with OG and LG (OR = 0.92 and 95% CI, 0.69−1.23; OR = 0.72 and 95% CI, 0.25−2.06) and (OR = 1.06 and 95% CI, 0.84−1.34; OR = 1.55 and 95% CI, 0.49−4.94). Moreover, subgroup analysis of subtotal and total gastrectomies in both RG *vs.* OG and RG *vs.* LG revealed that the scope of surgical dissection was not a positive factor to influence the comparative results of RG *vs.* OG or LG in surgery time, blood loss, hospital stay, lymph node harvest, morbidity, and mortality.

**Conclusions:**

This meta-analysis highlights that robotic gastrectomy may be a technically feasible alternative for gastric cancer because of its affirmative role in both subtotal and total gastrectomies compared with laparoscopic and open resections.

## Introduction

Gastric cancer is the fourth most common malignancy and second leading cause of cancer death in the world [1]. Surgical resection remains the only curative treatment option and open gastrectomy with lymphadenectomy took a leading position in the treatment of gastric cancer for a long time. Kitano *et al*. firstly reported the laparoscopy-assisted distal gastrectomy for gastric cancer in 1994 [2]. Since then, LG has been gradually spread worldwide [3]–[5].

Minimally invasive surgery represents a developing trend for its unique characteristics. However, conventional laparoscopic surgery itself, accompanied by some limitations such as instrument movement, amplification of hand tremor, two-dimensional imaging, and ergonomic discomfort for the surgeons. Robotic surgery, an emerging technology, was invented to overcome the disadvantages of conventional laparoscopic surgery in 1997 [6]. For robotic surgery, several robotic devices have been developed, but only the Da Vinci Surgical System was widely used [7]. To date, robotic surgery has been maturely adopted in many fields of advanced surgical procedures worldwide, especially for prostate cancer [8]. In the field of gastric cancer, robotic gastrectomy (RG) has been reported to be beneficial for patients, with less injury and also with compatible short-term oncologic outcomes to open gastrectomy (OG) or laparoscopic gastrectomy (LG) [9]–[20].

However, sample size, a single institution design and different appraise system of complications limited these studies to conclude objective result. To overcome these limitations, a meta-analysis of RG *vs.* OG or LG for gastric cancer was performed to determine the relative merits of RG for gastric cancer.

## Methods

### Publication Search

Three electronic databases (PubMed, EMBASE, and Cochrane Library) were searched (last search was updated on 01 June 2013, using the search terms: robotics OR robot PLUS gastrectomy PLUS cancer OR carcinoma OR adenocarcinoma OR malignancy PLUS open OR laparoscope). Article language was limited to English. All eligible studies were retrieved, and their bibliographies were checked for other relevant publications. Review articles and bibliographies of other relevant studies identified were hand-searched to identify additional eligible studies. Only published studies with full-text articles were included. When the same patient population was included in several publications, only the most recent or complete study was used in this meta-analysis.

### Inclusion Criteria

The inclusion criteria were as follows: (a) controlled studies of RG *vs*. LG or RG *vs*. OG for gastric cancer; (b) report on at least one of the outcome measures mentioned below; and (c) sufficient published data to estimate an odds ratio (OR) with 95% confidence interval (CI).

### Exclusion criteria

Abstracts, letters, editorials and expert opinions, reviews without original data, case reports and studies lacking control groups were excluded. The following studies or data were also excluded: (1) they reported on gastric surgery for benign lesions and gastrointestinal stromal tumor (GIST) and did not contain a distinct group of patients with gastric cancer, (2) the outcomes and parameters of patients were not clearly reported; (3) it was impossible to extract the appropriate data from the published results; and (4) there was overlap between authors or centers in the published literature.

### Quality Assessment

The methodological quality of the studies included was assessed. Jadad Scale and MINORS were usually used to assess the quality of RCTs and non-RCTs, respectively [21], [22].

### Data Extraction

Information was carefully extracted from all eligible studies by two of the authors (Zong L and Seto Y), according to the inclusion criteria listed above. The following information were collected from each study: first author’s surname, publication date, district, resection extent, reconstruction method, BMI index, TNM stage, study type, and total number of patients in RG group and OG group or LG group, respectively. We did not define a minimum number of patients for inclusion in our meta-analysis.

### Statistical Analysis

Odd ratios with 95% CI were used for the comparisons of dichotomous variables (e.g., morbidity, and mortality) between surgical methods according to the method of Woolf. Heterogeneity assumption was confirmed by the X^2^-based Q-test. A P-value greater than 0.10 for the Q-test indicated a lack of heterogeneity among the studies, therefore, the OR estimate for each study was calculated by the fixed-effects model (the Mantel-Haenszel method). Otherwise, the random-effects model (the DerSimonian and Laird method) was used. The significance of the pooled OR was determined by the Z-test and P>0.05 was considered statistically significant. Weighted mean difference (WMD) with 95% confidence intervals (95% CI) was calculated for continuous variables (e.g., operation time, and blood loss). WMD was pooled by using the inverse variance model. Sensitivity analyses were carried out to determine if modification of the inclusion criteria for this meta-analysis affected the final results. An estimate of potential publication bias was carried out using the funnel plot, in which the OR for each study was plotted against its log (OR). An asymmetric plot suggested possible publication bias. Funnel plot asymmetry was assessed using Egger’s linear regression test, a linear regression approach to measure funnel plot asymmetry on the natural logarithm scale of the OR. The significance of the intercept was determined by the t-test, as suggested by Egger (P<0.05 was considered representative of statistically significant publication bias). All statistical tests were performed with Review Manager Version 5.0 (The Cochrane Collaboration, Oxford, England).

## Results

### Study Characteristics

**Figure 1:**
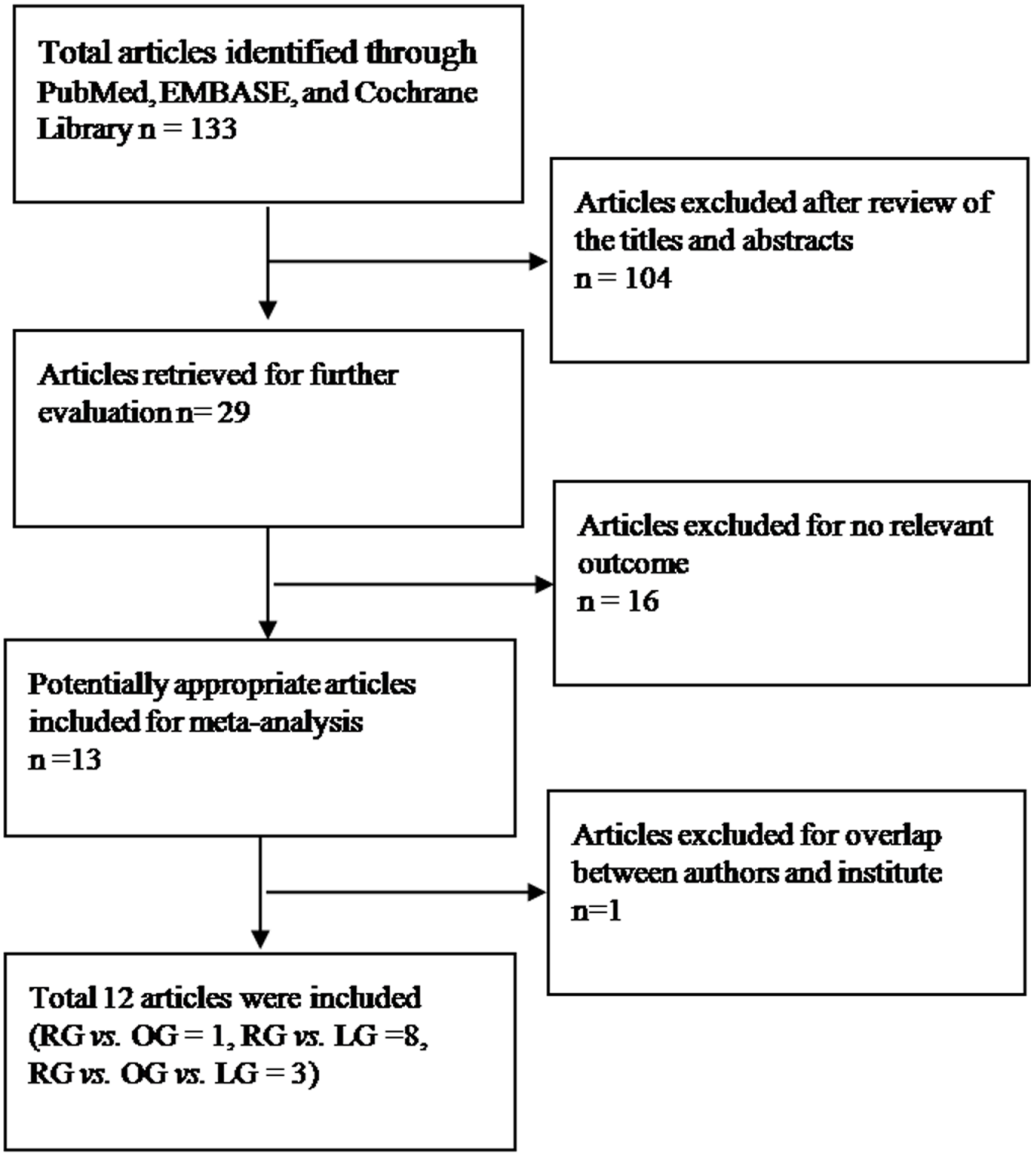
Flow chart of literature selection.

Of the 14 published pieces of literature [9]–[20], [23], 12 studies were eligible in this meta-analysis. Two studies published by the same team from the same institute within the same study interval were regarded as 1 trial, but both studies were included and shared the same study number because some separately published data was complementary [17], [23]. Hence, a total of 12 studies including 8493 patients were used in the pooled analyses. Table 1 lists the studies identified and their main characteristics. Of the 12 groups, sample size ranged from 39 to 5839 (Figure 1).

**Table 1:** Main characteristics of all studies included in the meta-analysis.

Study	StudyPeriod	District	Size	StudyGroup	Resectionextent	Reconstructionmethod	BMI	Stage	Studytype
Caruso *et al*	2011	Italy	149	RG/OG	Total/Subtotal12/17;37/83	NA	27±3/28±4	NA	Controlled
Eom *et al*	2012	Korea	92	RG/LG	Subtotal 30;62	NA	24.2/24.1	81/9/2	Controlled
Huang *et al*	2012	Taiwan	689	RG/LG/OG	Total/Subtotal7/32;7/57;179/407	B-I/B-II/Roux-en-Y	24.2±3.7/24. ±3.3/23±3.6	282/122/285	Controlled
Hyun *et al*	2012	Korea	121	RG/LG	Total/Subtotal9/29; 18/65	B-I/B-II/Roux-en-Y	23.8±2.6/23.8±2.9	97/14/10	Controlled
Kang *et al*	2012	Korea	382	RG/LG	Total/Subtotal16/84;37/245	B-I/B-II/Roux-en-Y	23.7±3.7/23.6±3.5	NA	Controlled
Kim *et al*	2010	Korea	39	RG/LG/OG	Subtotal 12;11;16	NA	21.3±3.4/25.3±2.5/25.2±1.9	27/9/3	Controlled
Kim *et al*	2012	Korea	5839	RG/LG/OG	Total/Subtotal109/158/1232;327/703/3309	B-I/B-II/Roux-en-Y	23.6±3.1/23.5±2.8/23.3±8.0	NA	Controlled
Park *et al*	2012	Korea	150	RG/LG	Subtotal 30;120	NA	NA	NA	Controlled
Pugliese *et al*	2010	Italy	64	RG/LG	Subtotal 16;48	NA	NA	NA	Controlled
Song *et al*	2009	Korea	40	RG/LG	Subtotal 20;20	NA	23.4±2.1/22.4±2.1	39/1/0	Controlled
Woo *et al*	2011	Korea	827	RG/LG	Total/Subtotal62/172; 108/481	B-I/B-II/Roux-en-Y	23.5±3/23.5±3	NA	Controlled
Yoon *et al*	2012	Korea	101	RG/LG	Total 36;65	NA	23.2±2.5/23.6±3.4	84/14/3	Controlled

### Robotic gastrectomy versus open gastrectomy

**Figure 2:**
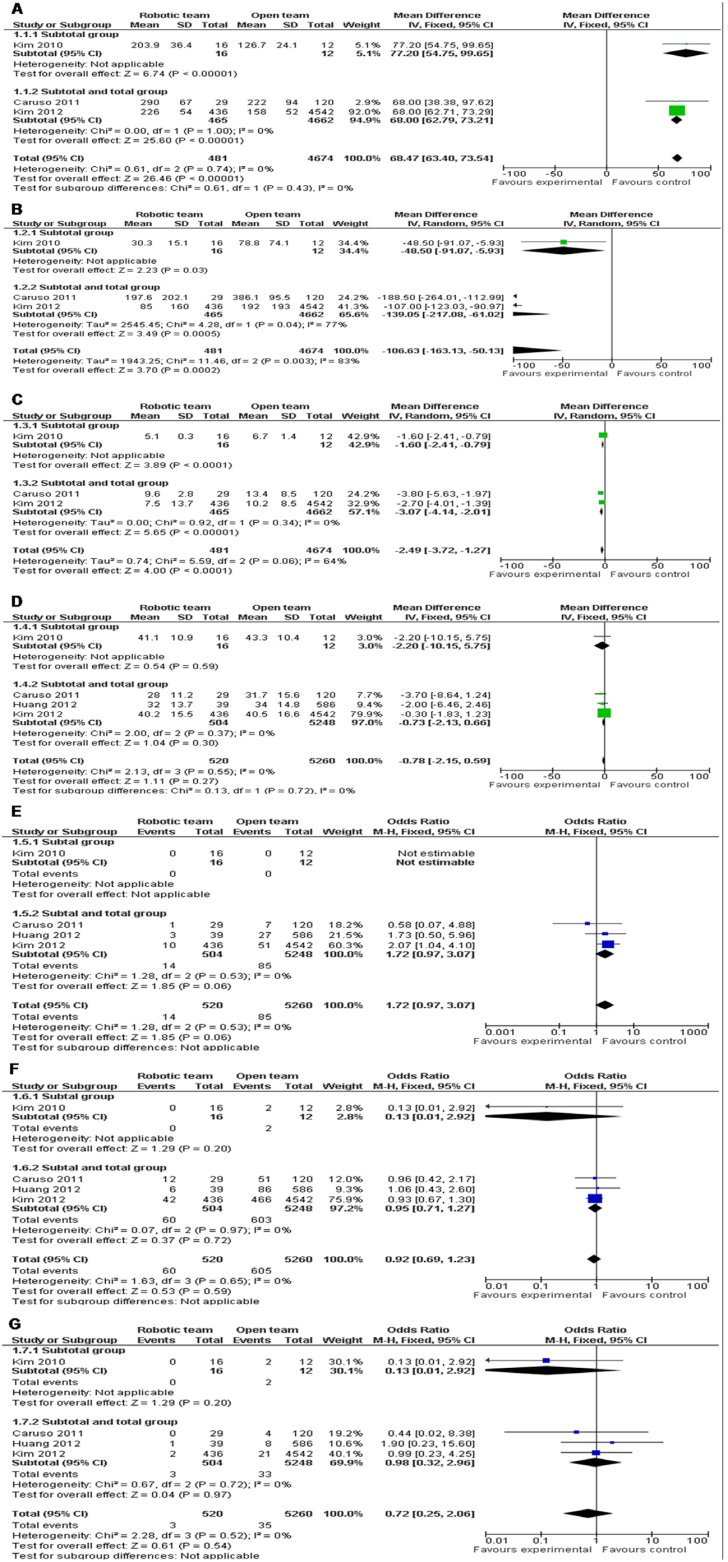
RG *vs.* OG: a) Operation time; b) Intraoperative blood loss; c) Hospital stay; d) Lymph node harvest; e) Anastomotic leakage; f) Morbidity; g) Mortality.

The mean operation time of RG was 68.47 minutes longer than OG, but intraoperative blood loss and hospital stay were significantly reduced by RG (WMD = 68.47 and 95% CI, 63.40−73.54; WMD = −106.63 and 95% CI, −163.13−−50.13; WMD = −2.49 and 95% CI, −3.72−−1.27). The difference of lymph node harvest between RG and OG was not statistically significant (WMD = −0.78 and 95% CI, −2.15−0.59). Moreover, Meta-analyses on morbidity and mortality indicated that there was no significant differences between RG and OG (OR = 0.92 and 95% CI, 0.69−1.23; OR = 0.72 and 95% CI, 0.25−2.06). Also, specifically for anastomotic leakage, no difference was observed between two groups (OR = 1.72 and 95% CI, 0.97−3.07). Subgroup analysis of subtotal gastrectomy, and subtotal and total gastrectomies for above parameters all showed a similar trend with the combined results (Table 2) (Figure 2).

**Table 2:** Meta-analyses results for robotic gastrectomy vs. open gastrectomy.

Parameters	Studies	SampleSize		Heterogeneity	OR orWMD	Effect95% CI	P
Operation time	3	481	4674	P = 0.74, I^2 = ^0%	68.47	63.40−73.54	P<0.00001
Subgroup of SG	1	16	12	NA	77.20	54.75−99.65	P<0.00001
Subgroup of SG and TG	2	465	4662	P = 1.00, I^2 = ^0%	68.00	62.79−73.21	P<0.00001
Intraoperative blood loss	3	481	4674	P = 0.003, I^2 = ^83%	−106.63	−163.13−−50.13	P = 0.0002
Subgroup of SG	1	16	12	NA	−48.50	−91.07−−5.93	P = 0.03
Subgroup of SG and TG	2	465	4662	P = 0.04, I^2 = ^77%	−139.05	−217.08−−61.02	P = 0.0005
Hospital stay	3	481	4674	P = 0.06, I^2 = ^64%	−2.49	−3.72−−1.27	P<0.0001
Subgroup of SG	1	16	12	NA	−1.60	−2.41−−0.79	P<0.0001
Subgroup of SG and TG	2	465	4662	P = 0.34, I^2 = ^0%	−3.07	−4.14−−2.01	P<0.00001
Lymph node harvest	4	520	5260	P = 0.55, I^2 = ^0%	−0.78	−2.15−0.59	P = 0.27
Subgroup of SG	1	16	12	NA	−2.20	−10.15−5.75	P = 0.59
Subgroup of SG and TG	3	504	5248	P = 0.37, I^2 = ^0%	−0.73	−2.13−0.66	P = 0.30
Anastomotic leakage	4	520	5260	P = 0.53, I^2 = ^0%	1.72	0.97−3.07	P = 0.06
Subgroup of SG	1	16	12	NA	NE	NE	NA
Subgroup of SG and TG	3	504	5248	P = 0.53, I^2 = ^0%	1.72	0.97−3.07	P = 0.06
Morbidity	4	520	5260	P = 0.65, I^2 = ^0%	0.92	0.69−1.23	P = 0.59
Subgroup of SG	1	16	12	NA	0.13	0.01−2.92	P = 0.20
Subgroup of SG and TG	3	504	5248	P = 0.97, I^2 = ^0%	0.95	0.71−1.27	P = 0.72
Mortality	4	520	5260	P = 0.52, I^2 = ^0%	0.72	0.25−2.06	P = 0.54
Subgroup of SG	1	16	12	NA	0.13	0.01−2.92	P = 0.20
Subgroup of SG and TG	3	504	5248	P = 0.72, I^2 = ^0%	0.98	0.32−2.96	P = 0.97

SG, subtotal gastrectomy; TG, total gastrectomy; NA, not applicable; NE, not estimable; OR, odds ratio; WED, weighted mean difference; CI, confidence interval.

### Robotic gastrectomy versus Laparoscopic gastrectomy

**Figure 3:**
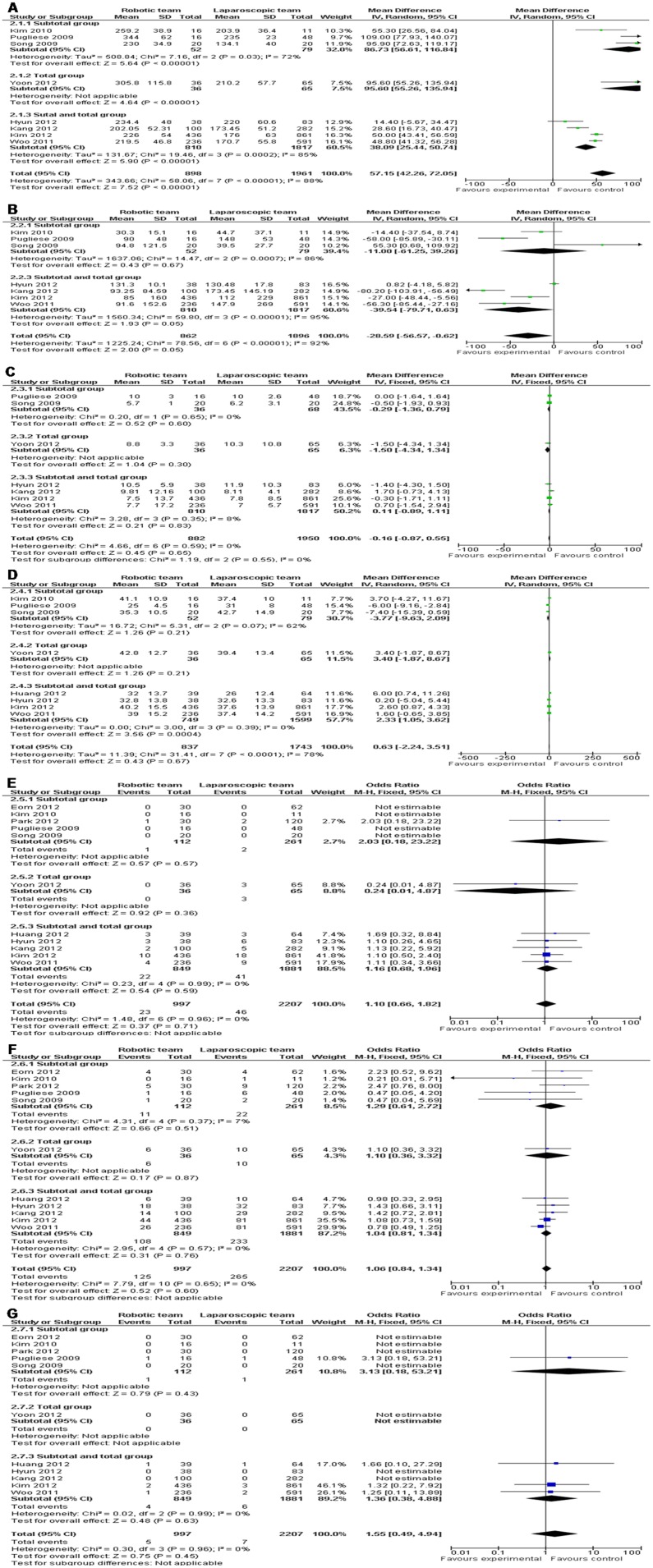
RG *vs.* LG: a) Operation time; b) Intraoperative blood loss; c) Hospital stay; d) Lymph node harvest; e) Anastomotic leakage; f) Morbidity; g) Mortality.

**Figure 4:**
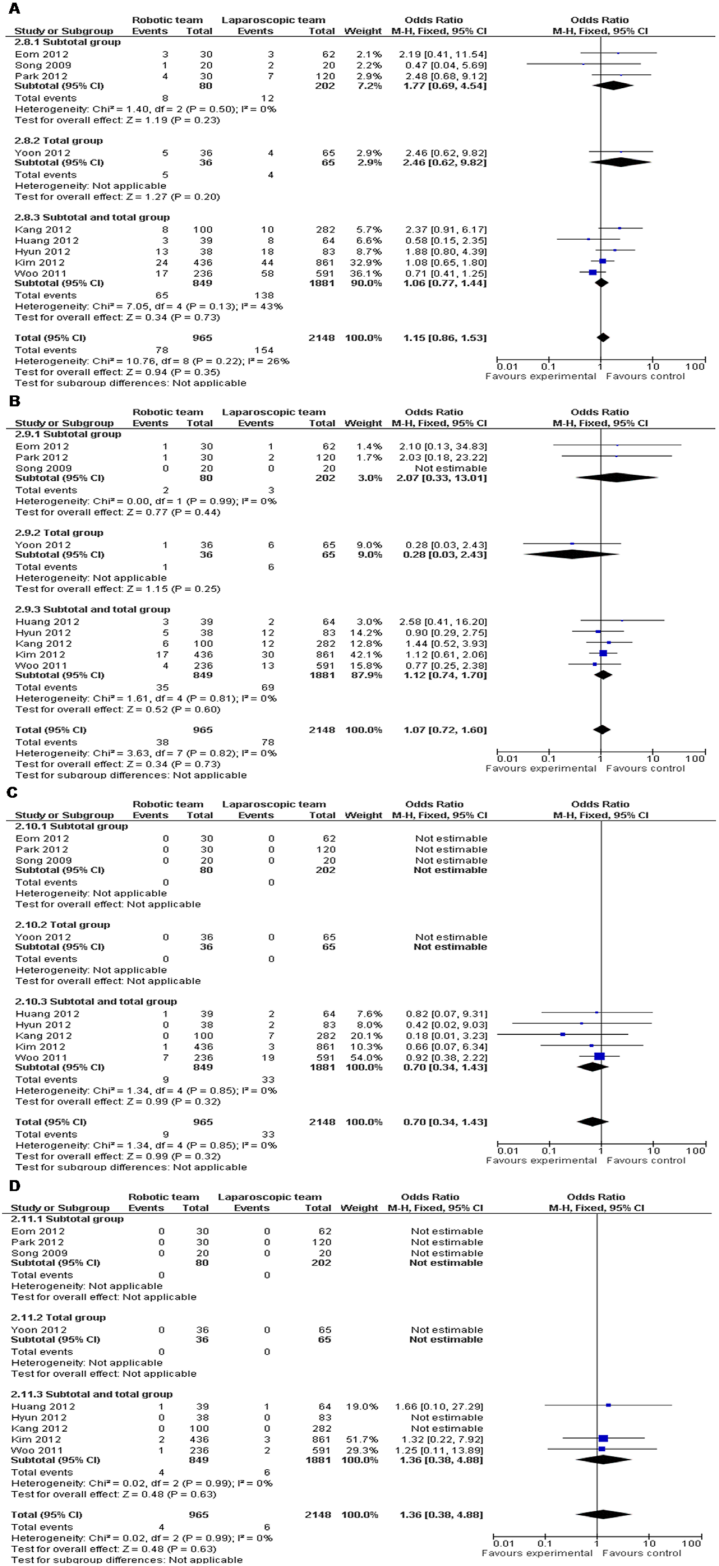
RG *vs.* LG: a) Clavien-Dindo grade I and II; b) Clavien-Dindo grade III; c) Clavien-Dindo grade IV; d) Clavien-Dindo grade V.

Operation time was significantly longer in RG compared with LG (WMD = 57.15 and 95% CI, 42.26−72.05). Both as the minimally invasive surgery, RG did not showed a priority in intraoperative blood loss (WMD =  −28.59 and 95% CI, −56.57−−0.62). As for postoperative hospital stay, there was no significant difference (WMD =  −0.16 and 95% CI, −0.87−0.55). In analysis of lymph node harvest, it did not attain statistical significance between RG and LG (WMD = 0.63 and 95% CI, −2.24−3.51). Further analysis revealed that RG did not carry additional postoperative morbidity, as well as anastomotic leakage, and mortality when compared with LG (OR = 1.06 and 95% CI, 0.84−1.34; OR = 1.10 and 95% CI, 0.66−1.82; OR = 1.55 and 95% CI, 0.49−4.94) (Table 3) (Figure 3). However, Meta-analysis on another surgical outcome evaluation system with Clavien-Dindo grades also did not show significant differences in any sub-divided grade. Subgroup analysis of subtotal gastrectomy, total gastrectomy, and subtotal and total gastrectomies was also performed for above parameters and no single subgroup showed a heterogeneous result with the combined one (Table 3) (Figure 4).

**Table 3:** Meta-analyses results for robotic gastrectomy vs. laparoscopic gastrectomy.

Parameters	Studies	SampleSize		Heterogeneity	OR orWMD	Effect95% CI	P
Operation time	8	898	1961	P<0.00001, I^2 = ^88%	57.15	42.26−72.05	P<0.00001
Subgroup of SG	3	52	79	P = 0.03, I^2 = ^72%	86.73	56.61−116.84	P<0.00001
Subgroup of TG	1	36	65	NA	95.60	55.26−135.94	P<0.00001
Subgroup of SG and TG	4	810	1817	P = 0.0002, I^2 = ^85%	38.09	25.44−50.74	P<0.00001
Intraoperative blood loss	7	862	1896	P<0.00001, I^2 = ^92%	−28.59	−56.57−−0.62	P = 0.05
Subgroup of SG	3	52	79	P = 0.0007, I^2 = ^86%	−11.00	−61.25−39.26	P = 0.67
Subgroup of SG and TG	4	810	1817	P<0.00001, I^2 = ^95%	−39.54	−79.71−0.63	P = 0.05
Hospital stay	7	882	1950	P = 0.59, I^2 = ^0%	−0.16	−0.87−0.55	P = 0.65
Subgroup of SG	2	36	68	P = 0.65, I^2 = ^0%	−0.29	−1.36−0.79	P = 0.60
Subgroup of TG	1	36	65	NA	−1.50	−4.34−1.34	P = 0.30
Subgroup of SG and TG	4	810	1817	P = 0.35, I^2 = ^8%	−0.11	−0.89−1.11	P = 0.83
Lymph node harvest	8	837	1743	P<0.0001, I^2 = ^78%	0.63	−2.24−3.51	P = 0.67
Subgroup of SG	3	52	79	P = 0.07, I^2 = ^62%	−3.77	−9.63−2.09	P = 0.21
Subgroup of TG	1	36	65	NA	3.40	−1.87−8.67	P = 0.21
Subgroup of SG and TG	4	749	1599	P = 0.39, I^2 = ^0%	2.33	1.05−3.62	P = 0.0004
Anastomotic leakage	11	997	2207	P = 0.96, I^2 = ^0%	1.10	0.66−1.82	P = 0.71
Subgroup of SG	5	112	261	NA	2.03	0.18−23.22	P = 0.57
Subgroup of TG	1	36	65	NA	0.24	0.01−4.87	P = 0.36
Subgroup of SG and TG	5	849	1881	P = 0.99, I^2 = ^0%	1.16	0.68−1.96	P = 0.59
Morbidity	11	997	2207	P = 0.65, I^2 = ^0%	1.06	0.84−1.34	P = 0.60
Subgroup of SG	5	112	261	P = 0.37, I^2 = ^7%	1.29	0.61−2.72	P = 0.51
Subgroup of TG	1	36	65	NA	1.10	0.36−3.32	P = 0.87
Subgroup of SG and TG	5	849	1881	P = 0.57, I^2 = ^0%	1.04	0.81−1.34	P = 0.76
Mortality	11	997	2207	P = 0.96, I^2 = ^0%	1.55	0.49−4.94	P = 0.45
Subgroup of SG	5	112	261	NA	3.13	0.18−53.21	P = 0.43
Subgroup of TG	1	36	65	NA	NE	NE	NA
Subgroup of SG and TG	5	849	1881	P = 0.99, I^2 = ^0%	1.36	0.38−4.88	P = 0.63
Clavien-Dindo grade							
I+II	9	965	2148	P = 0.22, I^2 = ^26%	1.15	0.86−1.53	P = 0.35
Subgroup of SG	3	80	202	P = 0.50, I^2 = ^0%	1.77	0.69−4.54	P = 0.23
Subgroup of TG	1	36	65	NA	2.46	0.62−9.82	P = 0.20
Subgroup of SG and TG	5	849	1881	P = 0.13, I^2 = ^43%	1.06	0.77−1.44	P = 0.73
III	9	965	2148	P = 0.82, I^2 = ^0%	1.07	0.72−1.60	P = 0.73
Subgroup of SG	3	80	202	P = 0.99, I^2 = ^0%	2.07	0.33−13.01	P = 0.44
Subgroup of TG	1	36	65	NA	0.28	0.03−2.43	P = 0.25
Subgroup of SG and TG	5	849	1881	P = 0.81, I^2 = ^0%	1.12	0.74−1.70	P = 0.60
IV	9	965	2148	P = 0.85, I^2 = ^0%	0.70	0.34−1.43	P = 0.32
Subgroup of SG	3	80	202	NA	NE	NE	NA
Subgroup of TG	1	36	65	NA	NE	NE	NA
Subgroup of SG and TG	5	849	1881	P = 0.85, I^2 = ^0%	0.70	0.34−1.43	P = 0.32
V	9	965	2148	P = 0.99, I^2 = ^0%	1.36	0.38−4.88	P = 0.63
Subgroup of SG	3	80	202	NA	NE	NE	NA
Subgroup of TG	1	36	65	NA	NE	NE	NA
Subgroup of SG and TG	5	849	1881	P = 0.99, I^2 = ^0%	1.36	0.38−4.88	P = 0.63

SG, subtotal gastrectomy; TG, total gastrectomy; NA, not applicable; NE, not estimable; OR, odds ratio; WED, weighted mean difference; CI, confidence interval.

### Publication Bias

Begg’s funnel plot was performed to assess publication bias. The heterogeneity tests for comparing the 12 combined studies showed heterogeneity in some analyses such as operation time, blood loss and so on; however, when significant heterogeneity occurred among the studies, random-effects model was used.

## Discussion

Radical gastrectomy with lymphadenectomy has been widely applied in open surgery as standard surgical treatment for gastric cancer. Although minimally invasive surgery improves quality of life, it should be ensured that this technique does not increase morbidity and mortality [24]. With the developing of technique, minimally invasive surgery has gained a revolutionized application in general surgery from last century. But for gastric cancer, minimally invasive surgery experienced a controversy focusing on morbidity and mortality for a long time. Laparoscopic gastrectomy with limited lymphadenectomy is rapidly increasing and quickly admitted in early gastric cancer because of the mass and individual screening in Japan [25]. But the data was still incomplete to support the widespread use of laparoscopic gastrectomy for advanced gastric cancer in last decade [26].

Open gastrectomy with D2 lymphadenectomy is a technically demanding operation for advanced gastric cancer compared with D1, although there is the potential for appreciable morbidity and mortality [27], [28]. Therefore, the assessment in favor of D2 lymphadenectomy makes it an integral part of laparoscopic surgery for advanced gastric cancer. Recently strong evidence from a multi-center retrospective study of laparoscopic surgery over open surgery confirmed the therapeutic role of Laparoscopic gastrectomy in advanced gastric cancer [29].

Robotic surgery, as an innovation of laparoscopic surgery, might be a simpler way to expand the indications of minimally invasive surgery for gastric cancer. However, controlled prospective studies are needed to evaluate the role of robotics in the management of gastric cancer. Some studies have demonstrated that robotic total and subtotal gastrectomies with D2-lymphadenectomy are technically feasible and safe, with acceptable surgical and oncological short-term results [15], [30]–[32]. It is particularly notable that only a few reports have examined the technical feasibility of robotic surgery for gastric cancer till 2011 [9], [14], [17]–[19], and the number of patients included in these studies was too small to generalize its application for gastric cancer [14], [17], [18]. Recently some large sized studies have been conducted to evaluate the efficacy and safety of robotic gastrectomy for gastric cancer [11], [13], [15], [19]. But single comparison and conflict results limited them to conclude persuasible conclusions. However, those examined in the present study allowed meta-analyses to be performed, providing a better view of the safety and efficacy of RG in gastric cancer. In reality, it is difficult to conduct a high-quality RCT to evaluate a new surgical intervention because of some obstacles such as learning curve effects, ethical and culture resistance, and urgent or unexpected conditions during operation in surgical treatment. For these reasons, to include non-RCTs is an appropriate strategy to extend the source of evidence [33].

In the first part of RG versus OG, our analyses highlighted the advantage of RG in minimal injury because less intraoperative blood loss and shorter postoperative hospital stay were observed. But its complication in technique correspondently brought RG significantly longer operation time than OG. Further analyses of lymph node harvest, anastomotic leakage, morbidity, and mortality between RG and OG did not show significant differences. Although no controlled study for single total gastrectomy was included in subgroup analysis, we deduced that RG was feasible and safe in either subtotal gastrectomy or total gastrectomy compared with OG by similar evidences in subtotal and total mixed group and subtotal single group.

Continually, in comparison of RG and LG, we found it was similar in surgical injury for these two methods because of no significant difference in intraoperative blood loss. The disadvantage of longer surgical duration was also observed in RG, although significant heterogeneity existed. The heterogeneity might be caused by surgeons’ experience. However, it is important to stress that surgeons had got considerable experience of LG before RG, which helped them adapt quickly to the robotic procedure. Therefore, the effect of learning curve was limited in RG. Also, higher BMI might be another important factor to increase operation time and several reports described the association between gender and BMI as increased operation time [34], [35]. But Park *et al* thought that this factor could be overcome by surgeon’s expertise [36]. To explore the influence of BMI to our study, we made comparisons of BMI among three groups and no significant difference was observed (data not shown). Importantly, for analyses of lymph node harvest, anastomotic leakage, morbidity, and mortality, similar results were achieved between RG and LG in either subtotal gastrectomy or total gastrectomy. We also make a pooled analyses using Clavien-Dindo (C–D) classification. Still, no significant difference was observed. What’s far more important to limit the application of RG is the higher cost compared with LG. Due to the limited published study, meta-analysis for cost evaluation was not performed. But nevertheless, recent study by Park *et al* showed the total cost for RG was significantly higher than LG with a difference of €3189 [16].

In summary, we found that Robotic subtotal and total gastrectomies combined with lymphadenectomy are technically feasible and safe for gastric cancer, and can produce satisfying short-term postoperative outcomes. However, a weakness of present study was lack of randomized controlled studies included and significant heterogeneity was observed in operative time, intraoperative blood loss, length of hospital stay and lymph node harvest. In addition, total and subtotal gastrectomy was pooled together in most of included studies, which limited us to make a more precise conclusion. Also, economic value and long-term survival outcome are the mandatory appraisal index. Importantly, high-quality randomized controlled studies should be conducted to evaluate the role of robotic surgery for gastric cancer in future.

## Supporting Information

Checklist S1 (DOC)

Diagram S1 (DOC)
