# Supplementary material for: Efficacy Evaluation of Subtotal and Total Gastrectomies in Robotic Surgery for Gastric Cancer Compared with that in Open and Laparoscopic Resections: A Meta-Analysis
Source: PLoS One. 2014 Jul 28;9(7):e103312. doi: 10.1371/journal.pone.0103312 (PMC4113385; doi:10.1371/journal.pone.0103312)
Supplement: Diagram S1 — (DOC) [file pone.0103312.s002.doc]

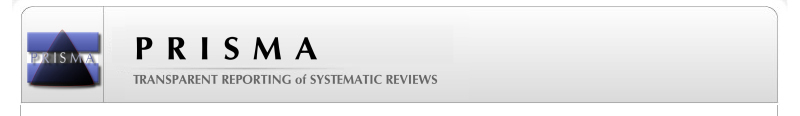
**PRISMA 2009 Flow Diagram**

**Screening**

**Included**

**Eligibility**

**Identification**

Records identified through database searching
(n = 115)

Additional records identified through other sources
(n =18)

Records after duplicates removed
(n = 112)

Records screened
(n = 29)

Records excluded
(n = 16 )

Full-text articles assessed for eligibility
(n =13)

Full-text articles excluded, with reasons
(n =1)

Studies included in qualitative synthesis
(n =12)

Studies included in quantitative synthesis (meta-analysis)
(n =12)
